# ACSL3 is a potential prognostic biomarker for immune infiltration in clear cell renal cell carcinoma

**DOI:** 10.3389/fsurg.2022.909854

**Published:** 2022-09-14

**Authors:** Chiyu Zhang, Honglin Hu, Ruizhen Huang, Gaomin Huang, Xiaoqing Xi

**Affiliations:** Department of Urology, The Second Affiliated Hospital of Nanchang University, Nanchang, China

**Keywords:** ACSL3, clear cell renal cell carcinoma, DNA methylation, immune cell component, survival value

## Abstract

**Objective:**

Long-chain acyl-coenzyme A synthases (ACSLs) catalyze the activation of fatty acid and are often dysregulated in malignancies. The purpose of this research was to figure out the ACSL3's prognostic value and mechanism in clear cell renal cell carcinoma (ccRCC).

**Methods:**

The expression of ACSL3 in ccRCC was investigated in this work using data from the GEO, TCGA, Oncomine and HPA databases. The expression differences of ACSL3 in the cell lines were further detected by qPCR and Western blot. GEPIA, MethSurv, cBioPortal, and the TIMER were used to perform survival and correlation analysis on ACSL3. GO and KEGG analyses were carried out in R using clusterProfiler and GOplot. Protein-protein interactions (PPI) are constructed from Strings website, and the results were visualized in Cytoscape software.

**Results:**

The expression level of ACSL3 was significantly reduced in ccRCC tissues, and its mRNA and protein expression were also significantly lower in both renal cancer cell lines. ACSL3 is significantly related to clinical stage, OS, DFS, DNA methylation, and immune-cell infiltration.

**Conclusion:**

Our findings demonstrated that data mining was capable of eliciting information on ACSL3 levels and its role in genetic regulatory pathways in ccRCC.

## Introduction

Renal cell carcinoma (RCC) is the sixth- and eighth-most frequent kind of cancer in males and females, respectively, with an anticipated seventy thousand new cases in the United States in 2020 (1). Furthermore, global RCC incidence rates have been rising over the last few decades (2). The average age of diagnosis is 60, and males are diagnosed at double the rate of women (3). Additionally, clear cell renal cell carcinoma (ccRCC) is the most prevalent kind of RCC, accounting for roughly 75% of all RCC cases (4). Despite the fact that ccRCC may be discovered early and effectively treated with surgical or ablative techniques, up to one-third of patients will present with or acquire metastases (5). This disease state is virtually always fatal, and it represents an important difference in the biology of ccRCC (6). In light of this, there is an urgent need for novel prognostic markers and therapeutic targets in ccRCC.

In addition to tumor cells, it is widely accepted that tumor tissue contains vascular cells, stromal cells, and immune cells, all of which play an important role in tumor growth and progression (7). In trials undertaken by CheckMate 214, immune checkpoint inhibitors (ICIs) have shown to be helpful for patients with advanced ccRCC (8). This demonstrates that cancer treatment has shifted its attention away from the tumor and toward the tumor microenvironment (TME), where tumor cells inhibit T lymphocytes (9). Numerous studies have shown that CD8 + T lymphocytes are abundant in the TME of RCC, although their relationship to therapy effect is debatable (10, 11).

A growing body of data suggests that dysregulation of fatty acid (FA) metabolism is linked to the tumorigenesis. Long-chain acyl-coenzyme A synthases (ACSLs) catalyze the activation of FA and are often dysregulated in malignancies (12). ACSL3′s *N*-terminal domain is in charge of controlling lipid droplets and FA absorption. Numerous cancers have ACSL3 overexpression, which is linked to a poor prognosis. It boosts oleic acid uptake and increases cell resilience to lipid oxidative damage. In an ACSL3-dependent way, oleic acid shields melanoma cells against ferroptosis and promotes cell distant metastasis (13). The prognosis of females with triple-negative breast cancer is significantly worsened by the presence of homozygous mutations in ACSL3 (14). It was shown that ACSL3 promotes pancreatic cancer growth by causing tumor cells to produce and secrete the profibrotic protein PAI-1 (15). Despite this, there has been no clear explanation regarding the function of ACSL3 in ccRCC.

## Materials and methods

### ACSL3 expression from oncomine analysis

ACSL3 mRNA expression in ccRCC was assessed using the Oncomine database (16) (http://www.oncomine.com). Oncomine is a combined online oncogene microarray database and data-mining platform that offers peer-reviewed, rigorous analytical methodologies as well as a sophisticated collection of analysis algorithms for calculating gene expression signatures. We focused on several ACSL3 studies including Higgins Renal (3 normal renal tissues vs. 23 ccRCC tissues), Lenburg Renal (9 normal colon tissues vs. 9 ccRCC tissues), Gumz Renal (10 normal renal tissues vs. 10 ccRCC tissues), and Beroukhim Renal (11 normal renal tissues vs. 27 ccRCC tissues). The ccRCC tissue was processed to quantify the expression level of ACSL3 in normal tissues.

### ACSL3 expression from TCGA and GEO databases

The ACSL3 expression data were obtained from the TCGA database (https://portal.gdc.cancer.gov/) and the GEO database (https://www.ncbi.nlm.nih.gov/geo/), which were analyzed using the limma package (17) of R software. The ACSL3 transcription in tumor tissues and renal tissues of ccRCC patients was evaluated using the Wilcoxon signed-rank test. The Illumina HiSeq 2000 RNA Sequencing platform was used to construct these gene expression profiles. The inclusion criteria for choosing GEO microarray datasets were as follows: ccRCC in humans; tumor and renal sample numbers. Finally, the GSE68417, GSE46699, and GSE167093 mRNA expression datasets were accessed, and raw data (TXT files) were obtained from the GEO repository. Platform annotation profiles were used to assign probes to genes. The quantile approach was used to standardize the microarray data.

### Immunohistochemical (IHC) staining analysis by HPA

Beginning in 2003 and using an array of cutting-edge omics techniques, the Human Protein Atlas (HPA) was a Swedish-based initiative tasked with cataloguing all human proteins across cells, tissues, and organs. HPA (18) (http://www.proteinatlas.org) was used to directly view immunohistochemical pictures of protein expression of distinct ACSL3 between normal and ccRCC samples in this investigation.

### Clinicopathological analysis of ACSL3 by UALCAN

UANLCAN (http://ualcan.path.uab.edu) is a web-based tool based on the TCGA (Cancer Genome Atlas) dataset (19). It may be used to compare the transcriptional expression of genes in benign and malignant lesions samples, as well as the transcriptional expression's relationship to relative clinicopathologic characteristics. UALCAN was employed in this work to examine the mRNA expressions of ACSL3 in ccRCC tissues and their relationship with clinicopathologic characteristics. The student's *t*-test was used to examine transcriptional expression differences, and *P* < 0.01 was judged statistically significant.

### Survival analysis of ACSL3

GEPIA can do survival analysis, correlation analysis, differential expression analysis, and look for genes that are related (19) (http://gepia.cancer-pku.cn/). This tool was used to perform survival analysis on ACSL3 and its related genes of ccRCC. On the basis of median mRNA expression levels, patients with ccRCC were divided into high and low expression groups. We used “survival” R package to perform univariate and multivariate Cox regression analysis to identify independent prognostic factors for ccRCC. Using the “timeROC” R package, a receiver operating characteristic curve (ROC) was produced to assess the predicting accuracy.

### ACSL3 mutation in ccRCC

Cancer genomics data sets may be interactively explored using the cBio Cancer Genomics Portal (20) (http://cbioportal.org), which is a free and open platform. We examined the ACSL3 mutation in the TCGA sample of ccRCC using cBioPortal. The “OncoPrint” page offered a broad picture of genetic changes in ACSL3 samples.

### MethSurv's DNA methylation data for ACSL3

The MethSurv database (21) (https://biit.cs.ut.ee/methsurv/) was used to analyze the DNA methylation sites of ACSL3 in TCGA. In addition, the prognostic value of CpG methylation in ACSL3 was evaluated.

### Immune infiltration analysis of ACSL3

TIMER (22) (https://cistrome.shinyapps.io/timer/) was used to assess ACSL3 expression in various cancers as well as immune infiltration levels. TIMER is a comprehensive online server that uses TCGA data to forecast immune cell infiltration in distinct tumor types and provides many modules of analysis on the quantity of immune infiltrates, including genes, survival, correlation, and other parameters. The correlation module in TIMER was also used to examine the correlations between ACSL3 expression and important molecules in common pathways.

### Analysis of functional enrichment and PPI network

Functional enrichment studies comprised GO and KEGG analyses, which were carried out using clusterProfiler (23) and GOplot (24) in R software. According to the size of -log10 (FDR), the differences between the FDR values of enrichment analysis are shown in histograms and bubble charts. The protein-protein interaction (PPI) networks are constructed from strings (25) (https://string-db.org/), and the results were visualized in Cytoscape software (26) (http://www.cytoscape.org/) with the minimum required interaction score of 0.700. Meanwhile, as an assessment tool, cytoHubba (27) was used to seek for hub genes.

### Cell culture

Normal renal epithelial cell lines (HK-2) and human renal cancer cell lines (786O and A498) were grown in RPMI1640 and MEM medium, respectively, throughout this study's experimental procedures. 10% heat-inactivated fetal bovine serum and 1% penicillin/streptomycin were included in all of the mediums. Additionally, all of the cells were cultured at 37 °C with 5% CO2.

### Quantitative PCR (qPCR)

The TRIzol reagent (Invitrogen) was used to extract total RNA from the cells. Following that, an RT-for-PCR kit was used to reverse transcribe 1 ug of the RNA into cDNA (Takara, Japan). Utilizing SYBR Green II, mRNA level quantification was carried out (Takara, Japan). The following is the order of the primers: ACSL3 forward primer 5′- GGCGTAGCGGTTTTGACA-3′, reverse primer 5′-CCAGTCCTTCCCAACAACGA-3′; GAPDH forward primer 5′- CAAGGTCATCCATGACAACTTT-3′, reverse primer 5′- GTCCACCACCCTGTTGCTGTAG -3′.

### Western blotting

To determine the amounts of ACSL3 protein expression, cells were disrupted using a RIPA buffer and protease inhibitors (Beyotime Biotechnology, China). On a 15% SDS-PAGE gel, 20 g of total protein was subsequently separated. The proteins were examined using antibodies specific for ACSL3 (20710–1-AP, Proteintech, China) (1:1,000) and GAPDH (60004–1-Ig, Proteintech, China) (1:20,000). Then, particular proteins were seen utilizing the AI600 Imaging System's approach (GE, USA).

## Results

### ACSL3 expression levels in various tumor types

**Figure 1:**
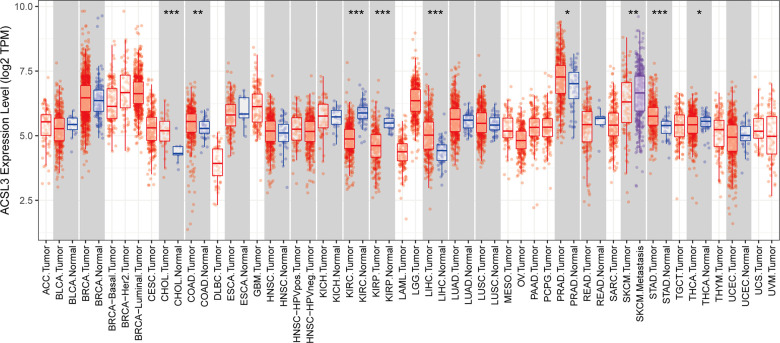
Analysis of the ACSL3 gene's differential expression in pan-cancers. *P < 0.05, **P < 0.01 and ***P < 0.001.

The Wilcoxon test was used to look for differences in the expression of ACSL3 in seventeen types of cancers and normal tissues, using the TIMER online database. ACSL3 expression was greater than that of matching normal tissue in the CHOL (bile duct cancer), COAD (colon cancer), LIHC (liver cancer), PRAD (prostate cancer), and STAD (stomach cancer), but was decreased in the KIRC (kidney clear cell carcinoma), KIRP (kidney papillary cell carcinoma), and THCA (thyroid cancer) (*P* < 0.05) (Figure 1).

### Expression of ACSL3 in ccRCC

**Figure 2:**
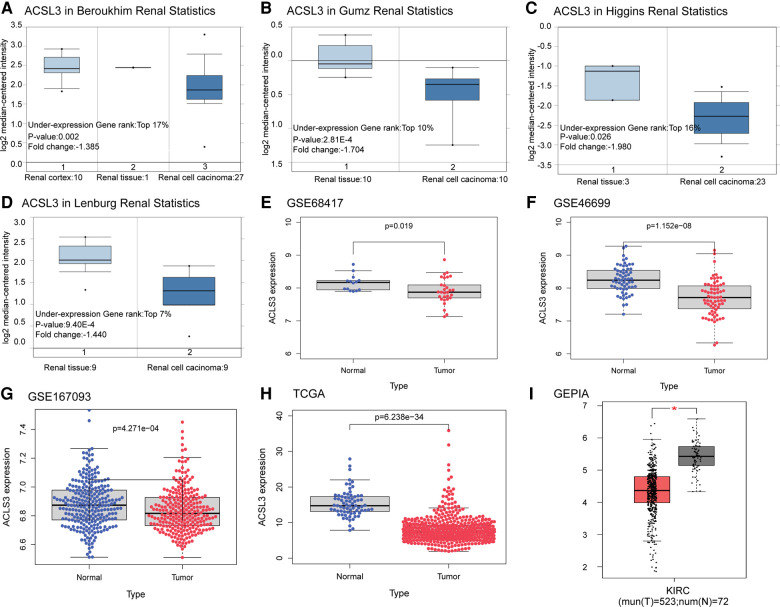
Differential expression analysis of ACSL3 gene. (**A–D**) Box plots depicting fold change and related *p* values based on Oncomine analysis (Higgins Renal, Lenburg Renal, Gumz Renal, Beroukhim Renal). (**E–G**) ACSL3 transcription in GSE68417, GSE46699, GSE167093. (**H**) ACSL3 transcription in TCGA. (**I**) ACSL3 transcription in GEPIA.

The ACSL3 transcription levels were compared across numerous research on ccRCC retrieved from the database. Using the Oncomine database, we first determined that ACSL3 mRNA expression was significantly lower in ccRCC tissues than in normal tissues when comparing much of the research (*P* < 0.05). As demonstrated in Figures 2A–D, ACSL3 mRNA expression was among the top 17%, with differences between ccRCC patients and normal tissues being no more than twofold. The bioinformatics study revealed that ACSL3 mRNA expression was considerably lower in ccRCC tumor tissues than in renal tissues retrieved from the TCGA, GEO, and GEPIA databases (Figures 2E–I).

**Figure 3:**
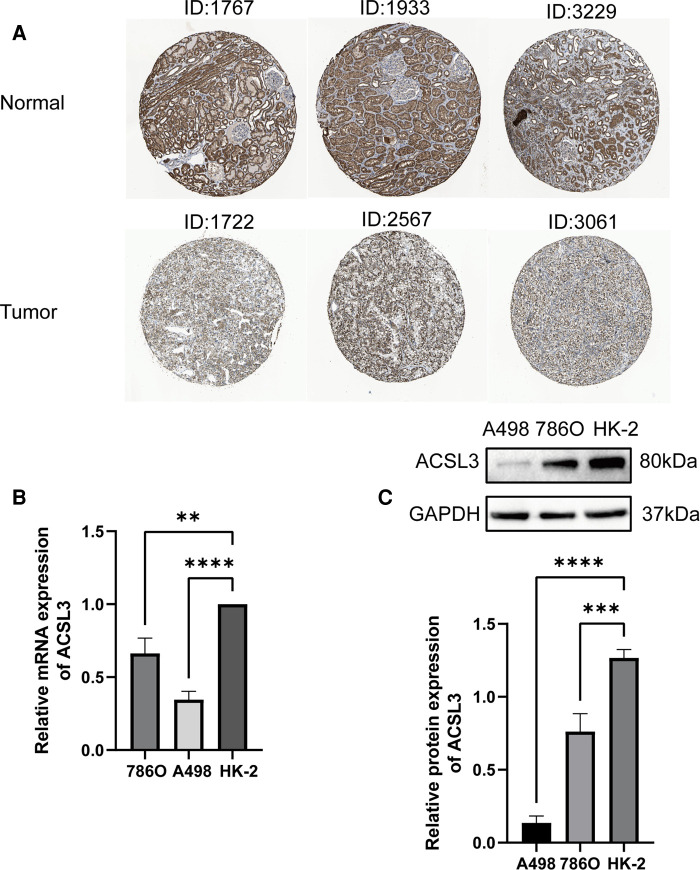
Protein and mRNA expression of ACSL3 in ccRCC. (**A**) Immunohistochemical staining pictures from ccRCC patients in the HPA database. Magnification,  × 100. (**B**) Quantitative real-time PCR of ACSL3 mRNA expression in normal renal cell line (HK-2) and renal cancer cell lines (7860 and A498). (**C**) Western blot detection of ACSL3 protein expression in cell lines.

It was shown that numerous patients have ACSL3 protein staining in their normal and ccRCC sections in the HPA database. In the HPA database, HPA011315 antibodies were used. ACSL3 was shown to be highly expressed in the cytoplasm and plasma membrane of normal cells and ccRCC tissue, respectively (Figure 3A). In addition, we analyzed the mRNA and protein expression of ACSL3 in both normal and cancerous renal cells. ACSL3 expression was lower in renal clear cell carcinoma cells, including 786O and A498, compared to normal HK-2 cells, as shown by the data (Figures 3B,C).

### ACSL3 expression in ccRCC subtypes

**Figure 4:**
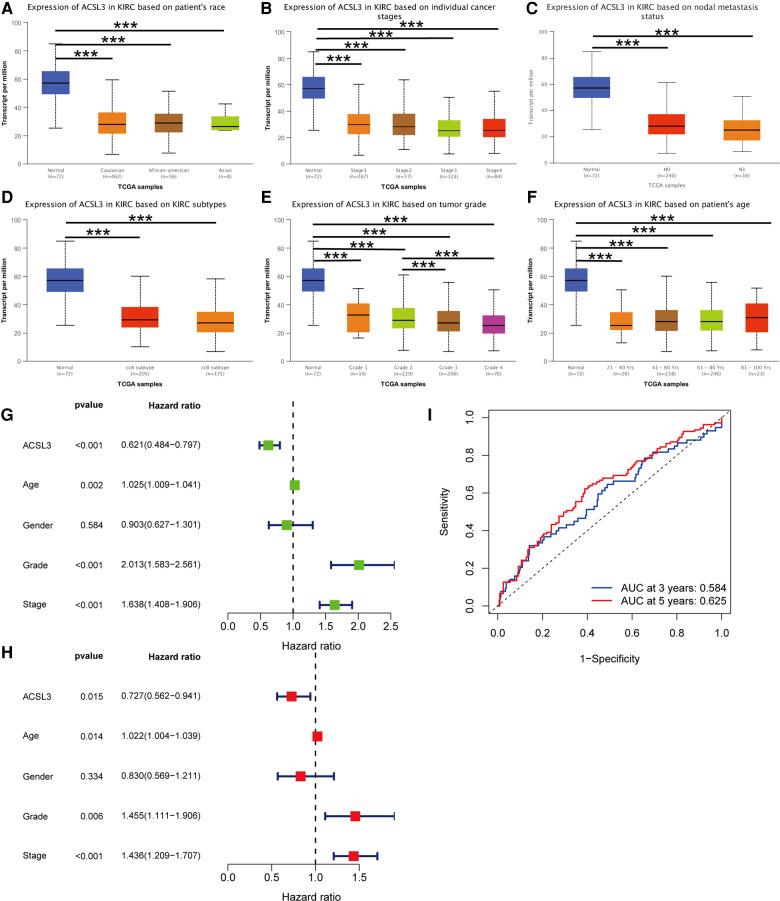
ACSL3 expression in subgroups of individuals with ccRCC. (**A**) A boxplot illustrating the expression of ACSL3 in patients with ccRCC of any ethnic origin. (**B**) A boxplot depicting the expression of ACSL3 in individuals with ccRCC at various stages (**C**) A boxplot illustrating ACSL3 expression in ccRCC patients with any level of nodal metastases. (**D**) A boxplot illustrating ACSL3 expression in ccRCC patients of various subtypes. (**E**) A boxplot depicting the expression of ACSL3 in individuals with ccRCC of different grades. (**F**) A boxplot depicting ACSL3 expression in ccRCC patients at different ages. (**G**) Univariate Cox regression analyses of overall survival. (**H**) Multivariate Cox regression analyses of OS. (**I**) The receiver operating characteristic (ROC) curve indicates that acsl3 expression may be able to predict overall survival. ***P < 0.001.

To further confirm ACSL3 specificity in ccRCC, we compared ACSL3 transcription levels in each group using multiple clinic features from the TCGA database, including stage, grade, ccRCC subtype, nodal metastatic status, patient race, and age. The results indicated that, in comparison to normal controls, high grade ccRCC patients retained a low level of ACSL3 transcription (Figures 4A–F). As a consequence, ACSL3 may be employed as a kidney biopsy marker for high-risk people with ccRCC.

### Lower ACSL3 expression predicts worse OS and DFS in ccRCC patients

**Figure 5:**
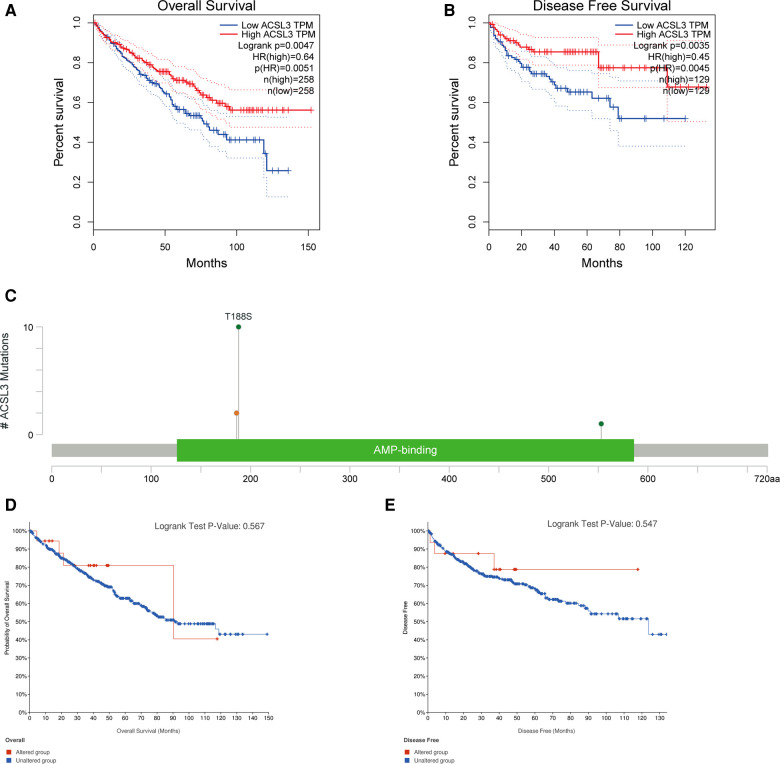
The ACSL3 mutation and prognosis in ccRCC. (**A,B**) KM survival curves for OS and DFS in patients with ccRCC. (**C**) The OncoPrint diagram illustrating the mutation in the ACSL3 gene. (**D,E**) OS and DFS survival curves comparing groups with and without the ACSL3 gene mutation.

Both univariate and multivariate Cox proportional risk analysis were conducted in this study. The findings demonstrated that the levels of ACSL3 expression, grade, and stages were independent predictive variables (Figures 4G,H). The 3-year and 5-year ROC curves' respective areas under the curves (AUC) were 0.584 and 0.625 (Figure 4I). The AUC value has some predictive power even if it is not extremely significant. Table 1 displays the clinical features of the samples from the two groups. In ccRCC, more examination of the relationship and impact of ACSL3 on patient survival was undertaken. This was accomplished *via* the usage of the GEPIA datasets. A lower level of ACSL3 was related to a shorter overall survival (OS, *P* = 0.0051) and disease-free survival (DFS, *P* = 0.0045) in ccRCC patients, demonstrating that down-regulated ACSL3 expression was a risk factor for a worse prognosis in ccRCC patients (Figures 5A,B).

**Table 1:** Correlation between ACSL3 expression and clinicopathological characteristics.

Covariates	Type	Total	ACSL3 > median	ACSL3 <= median	*P* value
Age	<=65	349 (65.73%)	165 (62.26%)	184 (69.17%)	0.1128
	>65	182 (34.27%)	100 (37.74%)	82 (30.83%)	
Gender	FEMALE	187 (35.22%)	71 (26.79%)	116 (43.61%)	1.00E–04
	MALE	344 (64.78%)	194 (73.21%)	150 (56.39%)	
	G1	14 (2.64%)	5 (1.89%)	9 (3.38%)	0.0109
	G2	228 (42.94%)	98 (36.98%)	130 (48.87%)	
	G3	206 (38.79%)	116 (43.77%)	90 (33.83%)	
	G4	75 (14.12%)	44 (16.6%)	31 (11.65%)	
	Unknow	8 (1.51%)	2 (0.75%)	6 (2.26%)	
Stage	Stage I	266 (50.09%)	115 (43.4%)	151 (56.77%)	0.0177
	Stage II	57 (10.73%)	30 (11.32%)	27 (10.15%)	
	Stage III	123 (23.16%)	73 (27.55%)	50 (18.8%)	
	Stage IV	82 (15.44%)	45 (16.98%)	37 (13.91%)	
	Unknow	3 (0.56%)	2 (0.75%)	1 (0.38%)	
T	T1	272 (51.22%)	117 (44.15%)	155 (58.27%)	0.0017
	T2	69 (12.99%)	38 (14.34%)	31 (11.65%)	
	T3	179 (33.71%)	107 (40.38%)	72 (27.07%)	
	T4	11 (2.07%)	3 (1.13%)	8 (3.01%)	
M	M0	421 (79.28%)	205 (77.36%)	216 (81.2%)	0.259
	M1	78 (14.69%)	44 (16.6%)	34 (12.78%)	
	Unknow	32 (6.03%)	16 (6.04%)	16 (6.02%)	
N	N0	239 (45.01%)	114 (43.02%)	125 (46.99%)	0.3743
	N1	16 (3.01%)	10 (3.77%)	6 (2.26%)	
	Unknow	276 (51.98%)	141 (53.21%)	135 (50.75%)	

### The ACSL3 mutation and prognosis in ccRCC

Most cancers, as well as their prognosis, are caused by gene mutations. We used cBioPortal to analyze the prevalence of ACSL3 mutations in 749 sequencing data from ccRCC patients in the TCGA collection. There were only 13 examples of ACSL3 mutations (1.6 percent), with 10 T188S, 2 × 186_splice and 1 D553N AA Change occurring in AMP-binding (Figure 5C). However, no significant change in the KM curves between the ACSL3 mutation group and the normal group was seen (*P* = 0.567) (Figures 5D,E). This study found that ACSL3 deficiency did not cause a bad outcome because of a mutation.

### DNA methylation and prognosis correlations

**Figure 6:**
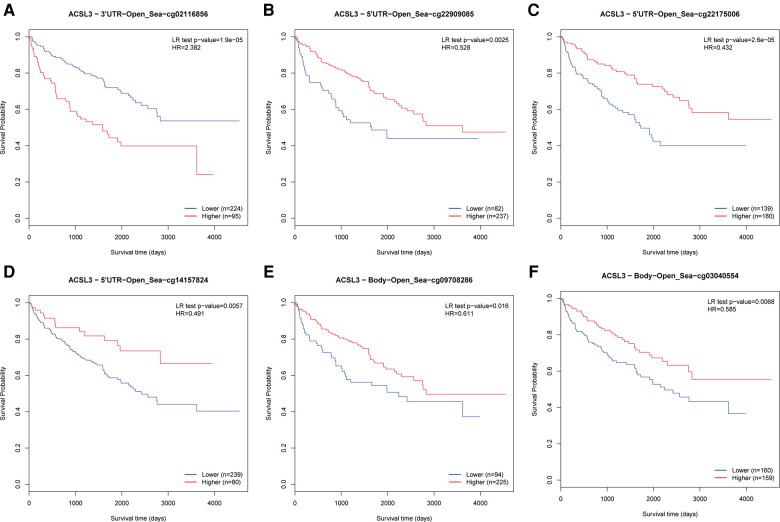
(**A–F**) Survival study of CpGs in ACSL3 using the Kaplan-Meier method.

The MethSurv tool was used to evaluate the DNA methylation levels of ACSL3, as well as the predictive significance of each single CpG. MethSurv findings showed that cg02116856 of ACSL3 had the highest DNA methylation (Figure 6A). Furthermore, the other 5 CpGs of ACSL3 were associated with ccRCC patient prognosis (Figures 6B–F).

### ACSL3 expression correlated with immune-cell infiltration in ccRCC

**Figure 7:**
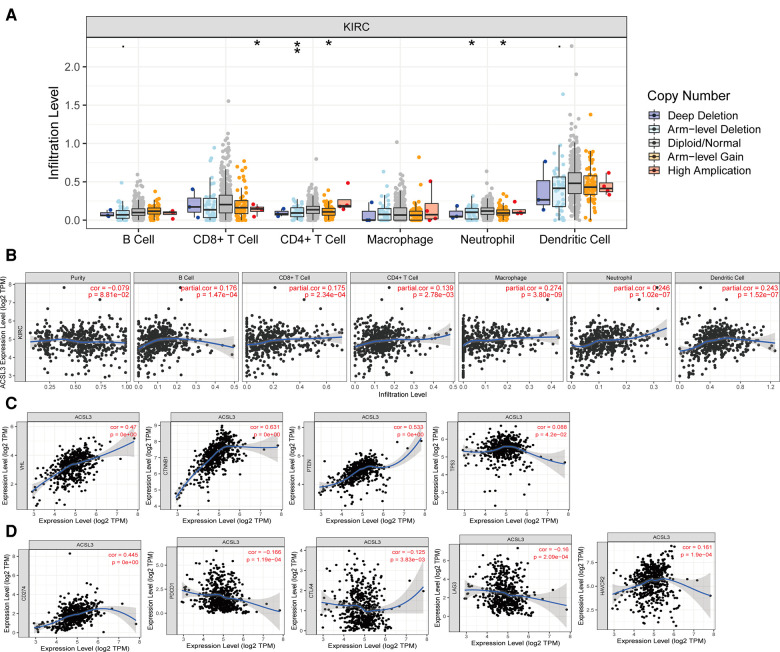
ACSL3 expression with immune infiltration in ccRCC by TIMER. (**A**) Correlation of ACSL3 copy number alterations (CAN) and immune cell infiltration. (**B**) Correlation between ACSL3 expression and immune cell infiltration. (**C**) Correlation between ACSL3 expression and important molecules involved in various pathways. (**D**) Correlation of ACSL3 expression with immune checkpoints molecules.

To begin, we examined the influence of ACSL3 copy number alterations (CNA) on the degrees of infiltration of various immune cells. The arm-level deletion of the ACSL3 gene significantly reduces the intensity of infiltration between CD4 + T cells (Figures 7A). Then, we studied the quantitative link between ACSL3 expression and immune cell infiltration in further detail. ACSL3 levels were shown to correlate positively with levels of infiltrating B cells (*r* = 0.176, *P* = 1.47e–04), CD8 + T cells (*r* = 0.175, *P* = 2.34e–04), CD4 + T cells (*r* = 0.139, *P* = 2.78e–03), macrophages (*r* = 0.274, *P* = 3.80e–09), neutrophils (*r* = 0.246, *P* = 1.02e–07), and dendritic cells (*r* = 0.243, *P* = 1.52e–07) (Figure 7B), indicating that ACSL3 may have an immuno-stimulating function.

We investigated the relationship between signaling pathway activation states and ACSL3 expression in greater detail since several signaling pathways have been linked to the initiation and development of ccRCC. We detected a positive correlation between the ACSL3 profile and the expression levels of VHL, PTEN, and TP53, all of which have been identified as tumor suppressor (Figure 7C). The TIMER database was utilized to assess the significance of ACSL3 and the currently available blocking compounds with superior therapeutic efficacy. In ccRCC, there was a substantial positive connection with PDL1 (CD274) and a weak positive correlation with PD1, CTLA4, LAG3, and TIM3 (Figure 7D).

### Enrichment analysis of ACSL3

**Figure 8:**
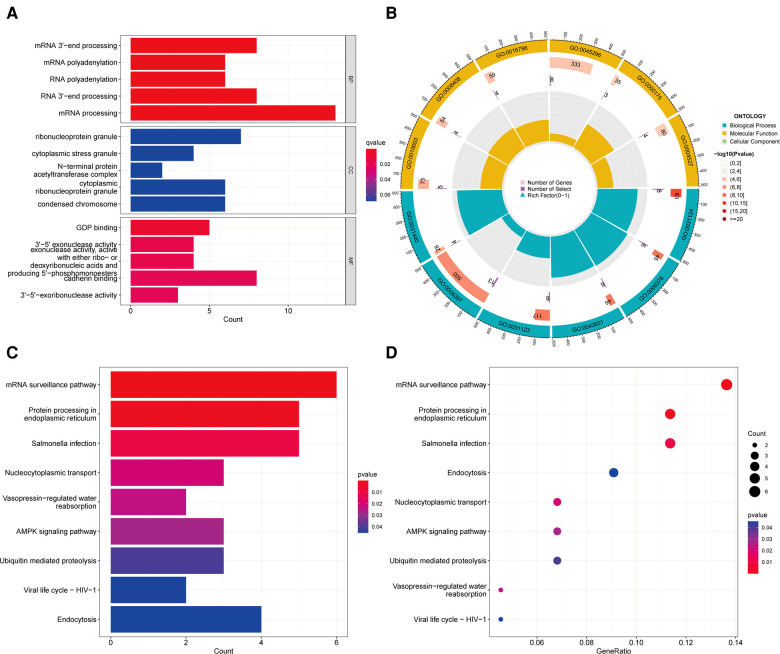
Go enrichment and KEGG pathway of ACSL3 in ccRCC. (**A**) Histogram of GO enrichment analysis. (**B**) Circle plot for GO enrichment analysis. (**C**) Histogram of KEGG pathway analysis. (**D**) Bubble plot for KEGG pathway analysis.

To further investigate ACSL3′s potential target genes in ccRCC, we utilized the GEPIA database to determine the top 100 ACSL3-related genes in ccRCC (Supplementary Table S1). We utilized R software to identify important KEGG pathways and to do enrichment analysis on all 100 genes. The ACSL3 significantly controlled the biological processes (BP) in ccRCC, including RNA polyadenylation, RNA 3′-end processing, and mRNA processing. The ACSL3 changes were substantially correlated with cellular components (CC), such as ribonucleoprotein granule, cytoplasmic stress granule, and cytoplasmic ribonucleoprotein granule. Furthermore, ACSL3 has a significant impact on molecular functions (MF) such as GDP binding, ribonucleoside binding, and exonuclease activity (Figures 8A,B; Supplementary Table S2). The KEGG analysis revealed an association between the functions of ACSL3 in ccRCC and mRNA surveillance pathway (Figures 8C,D; Supplementary Table S3).

### Functional networks in ccRCC

**Figure 9:**
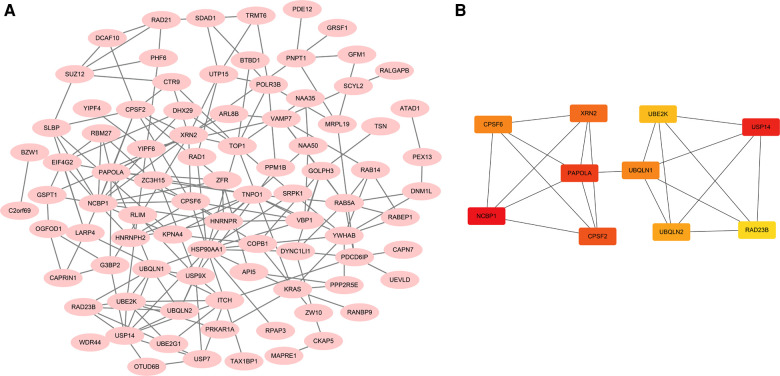
Functional analysis of ACSL3 in ccRCC. (**A**) A PPI network consisting of 100 ACSL3-related genes. (**B**) A PPI network with 10 hub genes.

**Figure 10:**
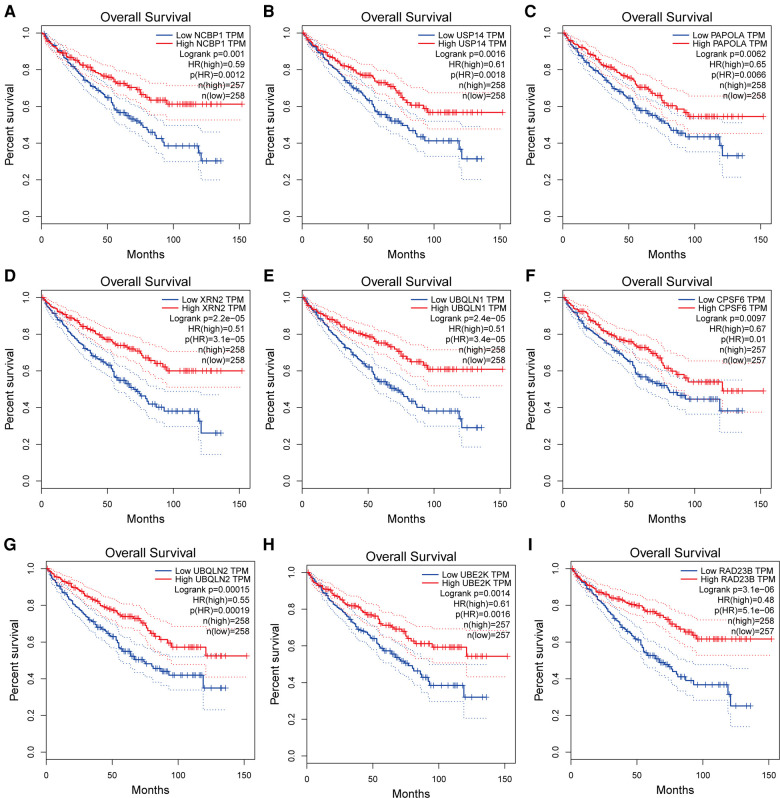
(**A–I**) Kaplan-Meier survival curves of hub genes in ccRCC patients.

STRING was used to create the PPI network, and the data were put into Cytoscape (Figure 9A). NCBP1, USP14, PAPOLA, CPSF2, XRN2, UBQLN1, CPSF6, UBQLN2, UBE2K, and RAD23B were identified as hub genes using the cytoHubba software (Figure 9B). We comprehensively analyzed the results, and found that most hub genes were strongly associated with ccRCC patients' survival (Figures 10A–I).

## Discussion

ccRCC is defined by relatively large malignant tumors that may readily develop into distant metastases, making early clinical detection a significant difficulty (28). Currently, B-ultrasound, CT, and MRI are utilized to diagnose ccRCC clinically. The risk of tumor recurrence after nephrectomy remains significant in individuals with confined malignancies (29). Because ccRCC is not amenable to radiation or chemotherapy, surgical excision has become the primary form of treatment (30). Patients with metastatic cancer, on the other hand, often miss the best period for surgery during therapy (31). As a result, it is critical to address the issue of identifying possible diagnostic and therapeutic biomarkers in ccRCC.

ACSL3 and CUB-domain containing protein 1 (CDCP1) work together to enhance TNBC cell metastasis by decreasing cell lipid droplet content and increasing oxidative phosphorylation (OXPHO) (32). Some researchers are also concerned about the close relationship between ACSL3 and cancer. ACSL3 has similar expression profiles in other cancer types, such as pancreatic cancer (15), breast cancer (32), lung cancer (33), and prostate cancer (34). ACSL3 overexpression has been discovered in a number of different types of cancer and is linked with a poor prognosis, the polar opposite of clear cell carcinoma. According to the findings of our present investigation, ACSL3 worked as a tumor suppressor gene in ccRCC in the following ways. Firstly, ACSL3 expression in ccRCC was substantially lower than normal tissue, especially with high grade ccRCC patients. Secondly, increased ACSL3 expression predicted higher survival outcomes, regardless of OS or DFS. Thirdly, the worse prognosis associated with decreased ACSL3 transcription is not the result of a genetic mutation. Finally, the 6 CpGs of ACSL3 were associated with ccRCC patient prognosis.

The adjuvant treatment for ccRCC has shifted from cytokine therapy to targeted therapy during the last three decades (35). Nevertheless, immune checkpoint blockade (ICB) has showed promise in treating ccRCC and is seen as a resurgence of ccRCC immunotherapy (36). In addition, the objective of this study was to demonstrate a connection between tumor immune cell infiltration and ACSL3 transcription in ccRCC. ACSL3 CNA was directly associated with immune cell infiltration levels, and subsequent research revealed that ACSL3 expression was shown to be positively connected with B cells, CD8 + T cells, CD4 + T cells, macrophages, neutrophils, and dendritic cell infiltration levels but negatively correlated with purity. In the tumor microenvironment, T cells undergo continuous antigen exposure, which can impair T cell function in layers, and eventually cause T cells to enter a dysfunctional state called “exhaustion” (37).

Numerous studies have shown that various classic signaling pathways, including VHL/HIF1α (38), PI3K/Akt/mTOR (39), HGF/met (40), MAPK (41) and Wnt/β- Catenin (42), are implicated in the promotion of ccRCC. ACSL3 expression has been discovered to be positively linked with tumor suppressor genes such as p53, Pten, and VHL. To put it another way, TRPV1 may inhibit ccRCC by activating these traditional signaling systems. However, some abnormal results were also shown in our analysis. ACSL3 has a favorable correlation with CTNNB1, a recognized tumor-promoting gene. The connection between ACSL3 and key pathways will be investigated in future study.

Furthermore, in order to fully understand how ACSL3 and immunity work together in the ccRCC, we looked into how ACSL3 interacts with immunosuppressive and immunostimulatory molecules in the body. Targeted inhibitors have been used in the area of tumor immunotherapy in recent years, after the discovery of immune checkpoint research, and have shown to have distinct therapeutic benefits. In 2011, the US Food and Drug Administration approved the humanized anti-CTLA4 antibody ipilimumab for the clinical treatment of metastatic melanoma (43). Meanwhile, the PD-1-PD-L1 axis antibody is suggested for the treatment of lung cancer, renal cell carcinoma, bladder cancer, liver cancer, and other cancer (44). We discovered that ACSL3 is substantially associated with the immune checkpoint inhibitors PD-1, PD-L1, CTLA4, LAG3, and HAVCR2 in renal clear cell carcinoma. Therefore, we speculate that ACSL3 might be utilized to predict the efficacy of immune-blocking treatment in tumor patients who are effective in immunotherapy monitoring.

To get a better understanding of the ACSL3 regulation network, we searched the GEPIA database for the top 100 ACSL3-related genes in ccRCC. After that, we built a PPI network graph of associated genes. This result indicates that NCBP1, USP14, PAPOLA, CPSF2, XRN2, UBQLN1, CPSF6, UBQLN2, UBE2K and RAD23B are the hub genes of the effective ACSL3 regulatory network. Except for CPSF2, we discovered that the survival rate of ccRCC patients is highly connected to the hub genes in this network. Functional enrichment analysis revealed that the top 100 ACSL3-related genes are mostly involved in the surveillance pathway and 3′-end processing of mRNA.

Our research does, however, have certain drawbacks and restrictions. First, additional clinical datasets should be included for external evaluation. Second, more experimental validation of the underlying molecular mechanism of ACSL3 in ccRCC is necessary. As a result, in our follow-up work, we will gather more and larger clinical samples and attempt to confirm the validity of our research with further outside trials.

## Conclusions

As shown by our results, ACSL3 expression was significantly downregulated in ccRCC tissues and renal cancer cell lines. ACSL3 expression was substantially linked with clinical stage, OS, DFS, DNA methylation, and immune cell infiltration, suggesting that it may be a target for clinical diagnosis, prognosis, and therapy of patients with ccRCC.

## Data Availability

The datasets presented in this study can be found in online repositories. The names of the repository/repositories and accession number (s) can be found in the article/**Supplementary material**.

## References

[1] Siegel RL Miller KD Jemal A. Cancer statistics. CA: Cancer J Clin. (2020) 70:7–30. 10.3322/caac.21590 31912902

[2] Ferlay J Colombet M Soerjomataram I Dyba T Randi G Bettio M Cancer incidence and mortality patterns in Europe: estimates for 40 countries and 25 major cancers in 2018. Eur J Cancer (Oxford, England: 1990). (2018) 103:356–87. 10.1016/j.ejca.2018.07.005 30100160

[3] Bray F Ferlay J Soerjomataram I Siegel RL Torre LA Jemal A. Global cancer statistics 2018: globocan estimates of incidence and mortality worldwide for 36 cancers in 185 countries. CA: Cancer J Clin. (2018) 68:394–424. 10.3322/caac.21492 30207593

[4] Lu J Zhu L Zheng LP Cui Q Zhu HH Zhao H Overexpression of ULK1 represents a potential diagnostic marker for clear cell renal carcinoma and the antitumor effects of SBI-0206965. EBioMedicine. (2018) 34:85–93. 10.1016/j.ebiom.2018.07.034 30078736 PMC6116477

[5] Jonasch E Gao J Rathmell WK. Renal cell carcinoma. BMJ. (2014) 349:g4797. 10.1136/bmj.g4797 25385470 PMC4707715

[6] Jonasch E Walker CL Rathmell WK. Clear cell renal cell carcinoma ontogeny and mechanisms of lethality. Nat Rev Nephrol. (2021) 17:245–61. 10.1038/s41581-020-00359-2 33144689 PMC8172121

[7] Wang Y Yang J Zhang Q Xia J Wang Z. Extent and characteristics of immune infiltration in clear cell renal cell carcinoma and the prognostic value. Transl Androl Urol. (2019) 8:609–18. 10.21037/tau.2019.10.19 32038957 PMC6987603

[8] Motzer RJ Tannir NM McDermott DF Arén Frontera O Melichar B Choueiri TK Nivolumab plus ipilimumab versus sunitinib in advanced renal-cell carcinoma. N Engl J Med. (2018) 378:1277–90. 10.1056/NEJMoa1712126 29562145 PMC5972549

[9] Sade-Feldman M Yizhak K Bjorgaard SL Ray JP de Boer CG Jenkins RW Defining T cell states associated with response to checkpoint immunotherapy in melanoma. Cell. (2018) 175:998–1013. 10.1016/j.cell.2018.10.038 30388456 PMC6641984

[10] Nakano O Sato M Naito Y Suzuki K Orikasa S Aizawa M Proliferative activity of intratumoral CD8(+) T-lymphocytes as a prognostic factor in human renal cell carcinoma: clinicopathologic demonstration of antitumor immunity. Cancer Res. (2001) 61:5132–6. 11431351

[11] Yao J Xi W Zhu Y Wang H Hu X Guo J. Checkpoint molecule PD-1-assisted CD8(+) T lymphocyte count in tumor microenvironment predicts overall survival of patients with metastatic renal cell carcinoma treated with tyrosine kinase inhibitors. Cancer Manag Res. (2018) 10:3419–31. 10.2147/cmar.S172039 30237743 PMC6138960

[12] Quan J Bode AM Luo X. ACSL Family: the regulatory mechanisms and therapeutic implications in cancer. Eur J Pharmacol. (2021) 909:174397. 10.1016/j.ejphar.2021.174397 34332918

[13] Ubellacker JM Tasdogan A Ramesh V Shen B Mitchell EC Martin-Sandoval MS Lymph protects metastasizing melanoma cells from ferroptosis. Nature. (2020) 585:113–8. 10.1038/s41586-020-2623-z 32814895 PMC7484468

[14] Jeong HM Kim RN Kwon MJ Oh E Han J Lee SK Targeted exome sequencing of Korean triple-negative breast cancer reveals homozygous deletions associated with poor prognosis of adjuvant chemotherapy-treated patients. Oncotarget. (2017) 8:61538–50. 10.18632/oncotarget.18618 28977883 PMC5617443

[15] Rossi Sebastiano M Pozzato C Saliakoura M Yang Z Peng RW Galiè M ACSL3-PAI-1 signaling axis mediates tumor-stroma cross-talk promoting pancreatic cancer progression. Sci Adv. (2020) 6. 10.1126/sciadv.abb9200 33127675 PMC7608806

[16] Rhodes DR Yu J Shanker K Deshpande N Varambally R Ghosh D ONCOMINE: a cancer microarray database and integrated data-mining platform. Neoplasia. (2004) 6:1–6. 10.1016/s1476-5586(04)80047-2 15068665 PMC1635162

[17] Ritchie ME Phipson B Wu D Hu Y Law CW Shi W Limma powers differential expression analyses for RNA-sequencing and microarray studies. Nucleic Acids Res. (2015) 43:e47. 10.1093/nar/gkv007 25605792 PMC4402510

[18] Thul PJ Åkesson L Wiking M Mahdessian D Geladaki A Ait Blal H A subcellular map of the human proteome. Science. (2017) 356. 10.1126/science.aal3321 28495876

[19] Chandrashekar DS Bashel B Balasubramanya SAH Creighton CJ Ponce-Rodriguez I Chakravarthi B UALCAN: a portal for facilitating tumor subgroup gene expression and survival analyses. Neoplasia. (2017) 19:649–58. 10.1016/j.neo.2017.05.002 28732212 PMC5516091

[20] Gao J Aksoy BA Dogrusoz U Dresdner G Gross B Sumer SO Integrative analysis of complex cancer genomics and clinical profiles using the cBioPortal. Sci Signal. (2013) 6:1. 10.1126/scisignal.2004088 PMC4160307 23550210

[21] Modhukur V Iljasenko T Metsalu T Lokk K Laisk-Podar T Vilo J. Methsurv: a web tool to perform multivariable survival analysis using DNA methylation data. Epigenomics. (2018) 10:277–88. 10.2217/epi-2017-0118 29264942

[22] Li T Fan J Wang B Traugh N Chen Q Liu JS TIMER: a web server for comprehensive analysis of tumor-infiltrating immune cells. Cancer Res. (2017) 77:e108–10. 10.1158/0008-5472.Can-17-0307 29092952 PMC6042652

[23] Yu G Wang LG Han Y He QY. Clusterprofiler: an R package for comparing biological themes among gene clusters. Omics. (2012) 16:284–7. 10.1089/omi.2011.0118 22455463 PMC3339379

[24] Walter W Sánchez-Cabo F Ricote M. GOplot: an R package for visually combining expression data with functional analysis. Bioinformatics. (2015) 31:2912–4. 10.1093/bioinformatics/btv300 25964631

[25] Szklarczyk D Gable AL Nastou KC Lyon D Kirsch R Pyysalo S The STRING database in 2021: customizable protein-protein networks, and functional characterization of user-uploaded gene/measurement sets. Nucleic Acids Res. (2021) 49:D605–d612. 10.1093/nar/gkaa1074 33237311 PMC7779004

[26] Reimand J Isserlin R Voisin V Kucera M Tannus-Lopes C Rostamianfar A Pathway enrichment analysis and visualization of omics data using: profiler, GSEA, cytoscape and enrichmentMap. Nat Protoc. (2019) 14:482–517. 10.1038/s41596-018-0103-9 30664679 PMC6607905

[27] Chin CH Chen SH Wu HH Ho CW Ko MT Lin CY. Cytohubba: identifying hub objects and sub-networks from complex interactome. BMC Syst Biol. (2014) 8(Suppl 4):S11. 10.1186/1752-0509-8-s4-s11 25521941 PMC4290687

[28] Satcher RL Zhang XH. Evolving cancer-niche interactions and therapeutic targets during bone metastasis. Nat Rev Cancer. (2022) 22(2):85–101. 10.1038/s41568-021-00406-5 34611349 PMC10281546

[29] Liang T Sang S Shao Q Chen C Deng Z Wang T Abnormal expression and prognostic significance of EPB41L1 in kidney renal clear cell carcinoma based on data mining. Cancer Cell Int. (2020) 20:356. 10.1186/s12935-020-01449-8 32760223 PMC7393885

[30] Ljungberg B Albiges L Abu-Ghanem Y Bensalah K Dabestani S Fernández-Pello S European association of urology guidelines on renal cell carcinoma: the 2019 update. Eur Urol. (2019) 75:799–810. 10.1016/j.eururo.2019.02.011 30803729

[31] Motzer RJ Jonasch E Boyle S Carlo MI Manley B Agarwal N NCCN guidelines insights: kidney cancer, version 1.2021. J Natl Compr Cancer Network. (2020) 18:1160–70. 10.6004/jnccn.2020.0043 PMC10191771 32886895

[32] Wright HJ Hou J Xu B Cortez M Potma EO Tromberg BJ CDCP1 drives triple-negative breast cancer metastasis through reduction of lipid-droplet abundance and stimulation of fatty acid oxidation. Proc Natl Acad Sci USA. (2017) 114:E6556–e6565. 10.1073/pnas.1703791114 28739932 PMC5559020

[33] Saliakoura M Reynoso-Moreno I Pozzato C Rossi Sebastiano M Galié M Gertsch J The ACSL3-LPIAT1 signaling drives prostaglandin synthesis in non-small cell lung cancer. Oncogene. (2020) 39:2948–60. 10.1038/s41388-020-1196-5 32034305 PMC7118021

[34] Migita T Takayama KI Urano T Obinata D Ikeda K Soga T ACSL3 Promotes intratumoral steroidogenesis in prostate cancer cells. Cancer Sci. (2017) 108:2011–21. 10.1111/cas.13339 28771887 PMC5623750

[35] Motzer RJ Hutson TE Tomczak P Michaelson MD Bukowski RM Rixe O Sunitinib versus interferon alfa in metastatic renal-cell carcinoma. N Engl J Med. (2007) 356:115–24. 10.1056/NEJMoa065044 17215529

[36] Motzer RJ Escudier B McDermott DF George S Hammers HJ Srinivas S Nivolumab versus everolimus in advanced renal-cell carcinoma. N Engl J Med. (2015) 373:1803–13. 10.1056/NEJMoa1510665 26406148 PMC5719487

[37] Thommen DS Schumacher TN. T cell dysfunction in cancer. Cancer Cell. (2018) 33:547–62. 10.1016/j.ccell.2018.03.012 29634943 PMC7116508

[38] Vuong L Kotecha RR Voss MH Hakimi AA. Tumor microenvironment dynamics in clear-cell renal cell carcinoma. Cancer Discov. (2019) 9:1349–57. 10.1158/2159-8290.Cd-19-0499 31527133 PMC6774890

[39] Jiang H Shi QQ Ge LY Zhuang QF Xue D Xu HY Selenoprotein M stimulates the proliferative and metastatic capacities of renal cell carcinoma through activating the PI3K/AKT/mTOR pathway. Cancer Med. (2019) 8:4836–44. 10.1002/cam4.2403 31274247 PMC6712446

[40] Ciamporcero E Miles KM Adelaiye R Ramakrishnan S Shen L Ku S Combination strategy targeting VEGF and HGF/c-met in human renal cell carcinoma models. Mol Cancer Ther. (2015) 14:101–10. 10.1158/1535-7163.Mct-14-0094 25381264 PMC4297225

[41] Hong B Zhou J Ma K Zhang J Xie H Zhang K TRIB3 promotes the proliferation and invasion of renal cell carcinoma cells via activating MAPK signaling pathway. Int J Biol Sci. (2019) 15:587–97. 10.7150/ijbs.29737 30745845 PMC6367588

[42] Piotrowska Ż Niezgoda M Młynarczyk G Acewicz M Kasacka I. Comparative assessment of the WNT/*β*-catenin pathway, CacyBP/SIP, and the immunoproteasome subunit LMP7 in various histological types of renal cell carcinoma. Front Oncol. (2020) 10:566637. 10.3389/fonc.2020.566637 33330038 PMC7717951

[43] Havel JJ Chowell D Chan TA. The evolving landscape of biomarkers for checkpoint inhibitor immunotherapy. Nat Rev Cancer. (2019) 19:133–50. 10.1038/s41568-019-0116-x 30755690 PMC6705396

[44] Gong J Chehrazi-Raffle A Reddi S Salgia R. Development of PD-1 and PD-L1 inhibitors as a form of cancer immunotherapy: a comprehensive review of registration trials and future considerations. J Immunother Cancer. (2018) 6(8). 10.1186/s40425-018-0316-z PMC5778665 29357948

